# The effect of Surround sound on embodiment and sense of presence in cinematic experience: a behavioral and HD-EEG study

**DOI:** 10.3389/fnins.2023.1222472

**Published:** 2023-09-07

**Authors:** Nunzio Langiulli, Marta Calbi, Valerio Sbravatti, Maria Alessandra Umiltà, Vittorio Gallese

**Affiliations:** ^1^Unit of Neuroscience, Department of Medicine and Surgery, University of Parma, Parma, Italy; ^2^Department of Philosophy “Piero Martinetti”, State University of Milan, Milan, Italy; ^3^Department of History, Anthropology, Religions, Arts and Performing Arts, Sapienza University of Rome, Rome, Italy; ^4^Department of Food and Drug, University of Parma, Parma, Italy; ^5^Italian Academy for Advanced Studies in America at Columbia University, New York, NY, United States

**Keywords:** HD-EEG, sense of presence, Surround sound, spatial audio, Alpha, Beta, ERD

## Abstract

Although many studies have investigated spectators' cinematic experience, only a few of them explored the neurophysiological correlates of the sense of presence evoked by the spatial characteristics of audio delivery devices. Nevertheless, nowadays both the industrial and the consumer markets have been saturated by some forms of spatial audio format that enrich the audio-visual cinematic experience, reducing the gap between the real and the digitally mediated world. The increase in the immersive capabilities corresponds to the instauration of both the sense of presence and the psychological sense of being in the virtual environment and also embodied simulation mechanisms. While it is well-known that these mechanisms can be activated in the real world, it is hypothesized that they may be elicited even in a virtual acoustic spatial environment and could be modulated by the acoustic spatialization cues reproduced by sound systems. Hence, the present study aims to investigate the neural basis of the sense of presence evoked by different forms of mediation by testing different acoustic space sound delivery (Presentation modes: Monophonic, Stereo, and Surround). To these aims, a behavioral investigation and a high-density electroencephalographic (HD-EEG) study have been developed. A large set of ecological and heterogeneous stimuli extracted from feature films were used. Furthermore, participants were selected following the generalized listener selection procedure. We found a significantly higher event-related desynchronization (ERD) in the Surround Presentation mode when compared to the Monophonic Presentation mode both in Alpha and Low-Beta centro-parietal clusters. We discuss this result as an index of embodied simulation mechanisms that could be considered as a possible neurophysiological correlation of the instauration of the sense of presence.

## Introduction

Cinema is a highly complex art form that combines visual and aural elements to create a cohesive and immersive experience. While the visual component of cinema has traditionally been the focus of both popular understanding and neuroscientific research (Heimann et al., 2014, 2019; Calbi et al., 2019), the role of sound in the cinematic experience has been largely overlooked. This bias toward the visual aspect can be attributed to a cultural tendency (Sterne, 2003) to prioritize sight over hearing as well as the fact that the human brain is wired to process visual information more efficiently than auditory information (Kitagawa and Ichihara, 2002; Sbravatti, 2019). Previous research has demonstrated that when participants are simultaneously presented with movies depicting facial emotions and emotional sounds (such as crying and laughing) that are incongruent with each other, the electromyography (EMG) signals recorded from their facial muscles are activated in accordance with the visual stimuli and not with the auditory stimuli (Sestito et al., 2013).

On the other hand, even if empirical research on the relationship between moving images and sounds in cinema is limited, some authors have suggested that sound could enhance the immersive qualities of the two-dimensional cinematic experience (moving images) by creating a sense of three-dimensional reality (Elsaesser and Hagener, 2015). This concept is also supported by the idea that Surround sound formats, such as 5.1 channel configurations, have the capability to envelop the viewer in a 360-degree auditory space as opposed to the traditional 180-degree visual space (DiDonato, 2010). Some studies have been conducted to investigate the relationship between Surround sound and the sense of presence (see below for a definition) in the cinematic experience. For example, Västfjäll found that 6-channel audio reproductions received a significantly higher presence and emotional realism scores than stereo (2-channels) and mono (1-channel) reproductions (Västfjäll, 2003). Kobayashi et al. (2015) examined the influence of spatialized sounds (reproduced by a 96-channel system) on the sense of presence in virtual environments by using both physiological and psychological measures. Results showed that the presence ratings for sounds in the spatialized sounds condition were higher. Furthermore, physiological measures such as heart rate and skin conductance level indicated that the sympathetic nervous system was activated to a greater extent by sounds in the spatialized sounds condition similar to the responses elicited during intrusions into peri-personal space in real-world scenarios (such as clapping near the participant) (Kobayashi et al., 2015).

In a 1997 study, Slater and Wilbur critically examined for the first time the often confused concepts of immersion and presence, suggesting a way to disambiguate their meanings. The two authors defined immersion as an objective property of the technological playback system and presence as the subjective psychological experience of feeling situated in a mediated environment (Slater and Wilbur, 1997). The spatial situational model framework suggests that the experience of presence in a mediated environment is achieved through a two-step process (Wirth et al., 2007). The first step is the construction of a spatialized mental model of the mediated environment, in which participants can perceive the environment as a space and locate themselves within it. Certain features of the mediated environment are particularly important for the formation of a spatialized mental model, and one of these features is Surround sound among others such as stereoscopy and field of view (Wirth et al., 2003). The second step is the embodiment of the mediated environment. Gallese proposes that “film experience and film immersion do not depend just on concepts and propositions, but rely on sensory-motor schemas, which get the viewer literally in touch with the screen, shaping a multimodal form of simulation, which exploits all the potentialities of our brain–body system” (Gallese, 2019), referring to embodied simulation, a cognitive process described as the ability to simulate the actions, emotions, and sensations of others by activating the same neural circuits that are used to perceive one's own experiences. This mechanism allows individuals to recognize the meaning of others' behaviors and experiences by directly relating to them through the activation of sensory-motor representations in the bodily format (Gallese, 2009). The neural substrate of the embodied simulation mechanism for actions corresponds to a particular functional group of neurons called “mirror neurons,” first discovered in area F5 of macaques during an intracortical recording of the premotor cortex that responds both during action execution and action observation (DiPellegrino et al., 1992). Mirror neurons allow for the internal representation of observed actions, which in turn facilitates understanding and imitation. According to Keysers et al., mirror neurons encode actions in an abstract manner, independent of the source of information (auditory or visual). This abstraction allows for multisensory integration, which is essential for generating meaningful representations and recognizing relevant actions within the environment (Keysers et al., 2003). In human beings, the mirror neuron mechanism is commonly associated with the mu rhythm typically recorded over sensorimotor centro-parietal cortical areas (Muthukumaraswamy and Johnson, 2004a,b; Muthukumaraswamy et al., 2004). The mu rhythm is an EEG measure of motor neuron activity considered to belong to the alpha band, generally ranging from 8 to 13 Hz, and the beta band, typically ranging between 14 and 32 Hz (Hari, 2006). When Gastaut and Bert (1954) initially observed the mu rhythm using EEG, they detected that this rhythm became less active, and there was an event-related desynchronization (ERD), when participants watched video clips of movements, but without exhibiting any visible motor movements themselves (Gastaut and Bert, 1954). Many subsequent studies observed a mu rhythm ERD, occurring during both voluntary movements, motor imagery, and action observation (Pfurtscheller et al., 1994; Toro et al., 1994; Pfurtscheller and Neuper, 1997; Neuper et al., 2006; Perry et al., 2010), and it has been proposed that this mu rhythm desynchronization represents activity in the mirror neuron system (e.g., Caetano et al., 2007; Perry and Bentin, 2009; Press et al., 2011).

The only study that investigated the effect of acoustic spatialization on the sense of presence using electroencephalography (EEG) was by Tsuchida et al. (2015). They used a surround sound reproduction system called BoSC (62 speakers), designed to simulate the presence of other individuals or objects by providing a highly realistic sound field, to deliver an acoustic stimulus under two experimental conditions: spatialized condition and monophonic condition (1-channel). EEG results showed that mu rhythm suppression occurred for action-related sounds but not for non-action-related sounds. Furthermore, this suppression was significantly greater in the Surround (62-channels) condition, which generates a more realistic sound field, than in the one-channel speaker condition. Additionally, the motor cortical activation for action-related sounds was influenced by the sense of presence perceived by the study participants as they perceived a significantly higher sound realism in the Surround condition (Tsuchida et al., 2015). It should be noted that this study had small participants and stimuli sample size, but only six action-related and non-action-related sounds were recorded and reproduced by an unconventional custom spatialized sound field system; hence, its results should be considered in light of the limitations of the study design. Further research with larger sample sizes and variegated stimuli is needed to fully understand the effect of acoustic spatialization on the sense of presence. Furthermore, the use of a standard surround sound reproduction system setup (such as 5.1-channel configurations) could ensure consistency and replicability compared to an unconventional setup.

Hence, this study aimed to investigate the time course and neural correlates of the sense of presence as evoked by different audio Presentation modes during cinematic immersion. We selected a diverse set of naturalistic stimuli, consisting of validated cinematic excerpts, which were presented to participants in different audio Presentation modes (Monophonic, Stereo, and Surround), while their neural and behavioral responses were measured. We first designed a behavioral experiment (Experiment 1) and subsequently a high-density electroencephalographic (HD-EEG) experiment (Experiment 2). Initially, in the context of the behavioral experiment, the sense of presence was rated by participants through explicit questions formulated to reflect its different aspects. The behavioral experiment was specifically designed to offer an initial investigation of the sense of presence with the aim to use results to guide the design of a subsequent EEG experiment. We hypothesized that participants exposed to the Surround presentation mode would report significantly higher subjective ratings compared to those exposed to the Monophonic and Stereophonic Presentation modes. Afterward, in the EEG experiment, we investigated the neural correlate of the sense of presence elicited by different acoustic Presentation modes. We hypothesized that the greater spatialization of sound in the Surround presentation mode, which more closely resembles a real-life hearing environment, would lead to a greater sense of embodiment as reflected by a higher ERD in the mu rhythm frequency band, compared to the Monophonic and Stereophonic presentation modes. This embodied simulation mechanism would be interpreted as a potential neurophysiological correlate of the rise of the sense of presence.

## Experiment 1

### Materials and methods

#### Participants

Thirty-two participants (*N* = 32, 14 men and 18 women, with a mean age *M* of 28.7 years and standard deviation SD of ±6.3, within a range of 22 to 42 years) were selected using an adaptation of the generalized listener selection (GLS) procedure described by Zacharov et al. (Mattila and Zacharov, 2001; Bech and Zacharov, 2006). The GLS procedures included six questionnaires, an audiometric test, and two screening tasks about loudness discrimination and localization of the sound source. For more information about GLS procedures, questionnaires, and descriptive statistics, see Supplementary material. Participants had a high education level (*M* = 15.5 years, SD = ± 2.3 years), had no prior history of neurological or psychiatric disorders, were right-handed as determined by the Italian version of the Edinburgh Handedness Inventory (Oldfield, 1971), had discriminative abilities of both loudness and sound source localization, had normal hearing acuity, and were “un-trained/naive subjects” as described in ITU-T Recommendation P.800 (ITU-R, 1996). All participants provided written informed consent to participate in the studies, which were approved by the local ethical committee “Comitato Etico Area Vasta Emilia Nord” and were conducted in accordance with the 1964 Declaration of Helsinki and its later amendments or comparable ethical standards (World Medical Association, 2013).

#### Acoustic apparatus

A silent audiometric cabin (IAC-Acoustics) 2 m high, 2.5 m wide, and 2.1 m deep was set up with a 5.1-channel surround sound reproduction system consisting of five APART MASK4C speakers (impedance 8 Ohms) and one APART SUBA165 sub-woofer (impedance 4 Ohms), all driven by a DENON AVR-X1600H amplifier. The participant was positioned at the center of the silent audiometric cabin, while the six speakers channels (“L” = left, “R” = right, “C” = center, “Ls” = left Surround, “Rs” = right Surround, “LFE” = low-frequency effects or sub-woofer) were positioned and oriented following the ITU-R BS.1116-1 recommendation so as to direct the sound to a central point that identified the reference listening position (ITU-R, 1997). Audio reproduction was room-corrected using the Audyssey software (Paul, 2009). Sound pressure levels (SPL) were recorded with a sound level meter (Gain Express, applied standard IEC651 type 2, type ANSI 2 SI 0.4) placed at the listening position, and the reproduction level was set below the hazardous hearing threshold (85 dB, A-weighted, for eight consecutive hours) defined and standardized by the National Institute for Occupational Health and Safety (NIOSH) in the ONE (Occupational Noise Exposure) recommendation (Murphy and Franks, 2002).

#### Stimuli

Twenty-seven cinematic excerpts (10 s long) without music and dialogues were chosen through an online validation experiment (see Supplementary material). We selected stimuli that had high dynamism, high emotional intensity, and negative emotional valence because these characteristics can elicit stronger arousing responses in the participants. Previous studies have demonstrated that negative audio-visual stimuli from feature films can increase arousal levels (Fernández-Aguilar et al., 2019). Our 27 stimuli were used in three Presentation modes: Surround, Stereo, and Monophonic for a total of 81 experimental stimuli, repeated twice. Stimuli were reproduced in the silent audiometric cabin by all six channels in the Surround reproduction mode, by “L” and “R” channels in the Stereo reproduction mode, and only by the “C” channel in the Monophonic reproduction mode. For more information about the stimuli selection procedure, see Supplementary material.

#### Procedure

Participants listened to 27 cinematic excerpts (10 s long) reproduced in three Presentation modes, played randomly twice for a total of 162 trials. The experiment was divided into three blocks of 54 trials each, with a break between blocks for a total experiment duration of ~45 m. Each trial consisted of a black fixation cross on a gray background (1.5 s), followed by the auditory stimulus presented for 10 s on a black screen. After viewing the stimulus, each time participants had 5 s to respond to two questions, randomly selected from a pool of four questions, on a Visual Analog Scale (VAS) from 0 to 100. The questions were formulated by the authors to measure four potential aspects of the cinematic immersion and sense of presence induced by the sound excerpt: Enjoyment (EN)—“How much did you like the scene?”; Emotional Involvement (EI)—“How much did you feel emotionally involved?”; Physical Immersion (PI)—“How much did you feel physically immersed?”; and Realism (RE)—“How realistic did you judge the scene?” (for more information, see Supplementary material). Before the experiment, we trained participants with six trials, two per each Presentation mode, using stimuli previously excluded through the validation process. A gray background was used as an inter-trial interval (ITI) with a duration of 3.5 s. At the end of the experimental session, the participant was asked to fill out the Film Immersive Experience Questionnaire (F-IEQ) (Rigby et al., 2019). For descriptive statistics, see Supplementary material. Stimuli were presented with MATLAB extension Psychtoolbox-3 (Brainard, 1997).

#### Analysis

In order to investigate whether Enjoyment (EN), Emotional Involvement (EI), Physical Immersion (PI), and Realism (RE) were modulated by the experimental conditions, a linear mixed-effect analysis was performed. Following a hierarchical approach, we initially created a simple model using one parameter, and we progressively added others with the aim to evaluate whether their inclusion improved model fit. Likelihood ratio tests, Akaike Information Criterion (AIC), and Bayesian Information Criterion (BIC) were used to rigorously choose which parameters improved model fit. We entered participants' scores as dependent variables, and Questions (EN, EI, PI, and RE, respectively) and Presentation modes (three levels: Surround, Stereo, and Monophonic) as independent fixed variables. Participants were included as a random intercept and Presentation mode as a random slope. This approach accounted for the within-subject and between-subject variability in the data. Outliers were identified and excluded from the analysis based on the standardized model residuals and a threshold value of Cook's distance (threshold = 1). *Post-hoc* tests were conducted using Tukey's correction for multiple comparisons and Kenward–Roger degrees-of-freedom approximation method. Statistical analyses were performed using R software (R Core Team, 2022), lme4 (Bates et al., 2015), effects (Fox and Weisberg, 2019), and emmeans (Lenth, 2022) packages. For data plotting, we used the ggplot2 (Wickham, 2016) package.

### Results

The model explained 85% of the variance in the dependent variable taking into account the random effects ($R_{\text{m}}^{2}$ = 0.22; $R_{\text{c}}^{2}$ = 0.85). The model revealed a significant main effect of Presentation modes [$\chi_{\left(2\right)}^{2}$ = 65.16, *p* < 0.001), showing that participants attributed significantly higher absolute scores when stimuli were presented in the Surround Presentation mode than when they were presented in the Stereo Presentation mode [*t*_(31)_ = 7.76, *p* < 0.001] or in the Monophonic Presentation modes [*t*_(30.9)_ = 7.76, *p* < 0.001; Surround: *M* = 59.44, CIs = 54.93, 63.95; Stereo: *M* = 51.4, CIs = 47.35, 55.44; Monophonic: *M* = 41.15, Cis = 36.01, 46.29]. At the same time, participants attributed significantly higher scores when stimuli were presented in the Stereo Presentation mode than when they were presented in the Monophonic Presentation mode [*t*_(31)_ = 6.26, *p* < 0.001].

The model also revealed a significant main effect of Question [$\chi_{\left(3\right)}^{2}$ = 71.57, *p* < 0.001] showing that participants attributed higher scores on Realism than on Enjoyment [*t*_(31)_ = 3.63, *p* < 0.01], Emotional Involvement [*t*_(31)_ = 5.7, *p* < 0.001], and Physical Immersion [*t*_(31)_ = 3.23, *p* < 0.01; EI: *M* = 46.33, CIs = 41.56, 51.11; EN: *M* = 47.53, CIs = 41.34, 53.71; PI: *M* = 52.21, CIs = 48.29, 56.14; RE: *M* = 56.57, CIs = 53.31, 59.84]. In addition, participants attributed higher scores to Physical Immersion than to Emotional Involvement [*t*_(30.9)_ = 5.55, *p* < 0.001].

**Figure 1:**
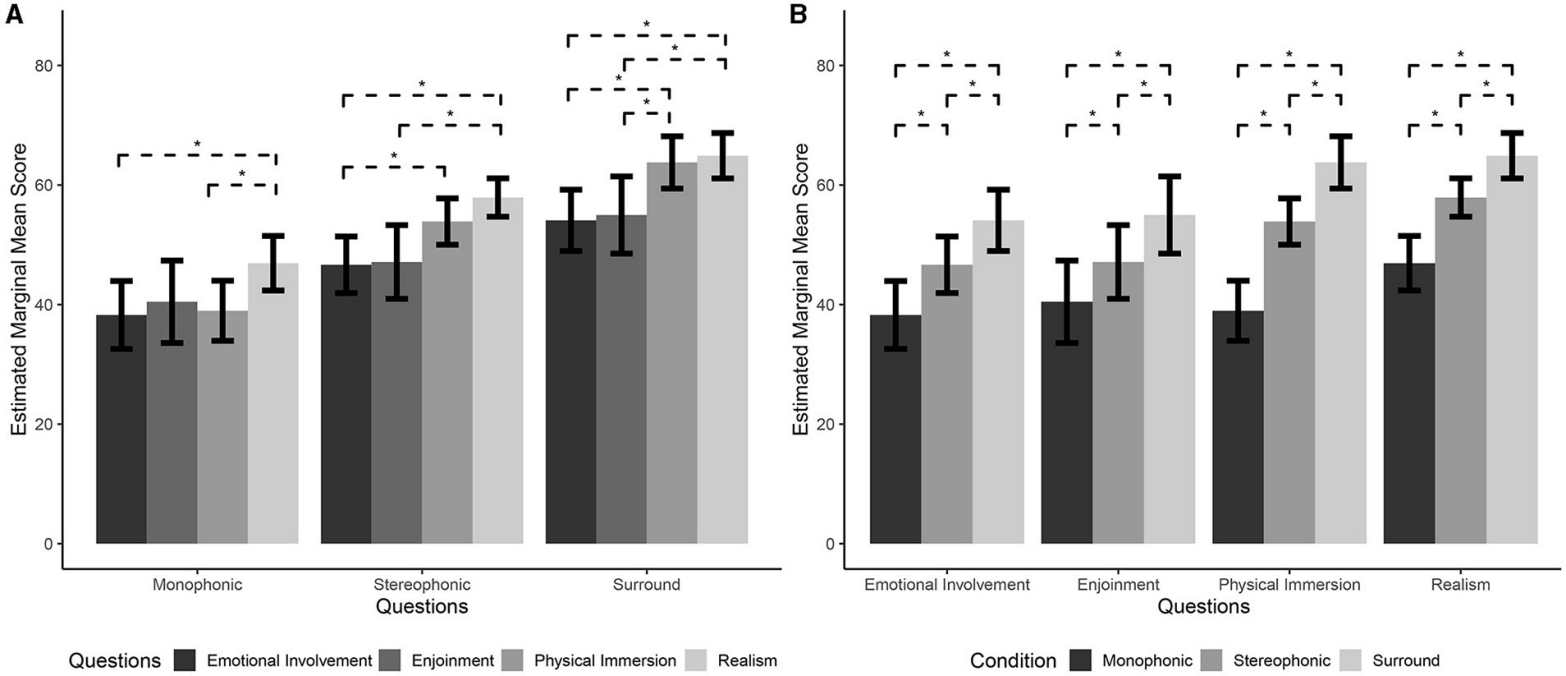
**(A)** Interaction effect *post-hoc* pairwise comparisons by Presentation modes. **(B)** Interaction effect *post-hoc* pairwise comparisons by Question. Error bars represent 95% confidence intervals of the mean (CI); asterisks (^*^) represents *p* < 0.05.

Additionally, the model revealed significant Presentation modes^*^Question interaction [$\chi_{\left(6\right)}^{2}$ = 269.36, *p* < 0.001]. Interaction *post-hoc* comparisons showed that in Monophonic Presentation mode (Figure 1A), participants attributed significantly higher scores on Realism than on Emotional Involvement [*t*_(34.6)_ = 4.69, *p* < 0.001] and Physical Immersion [*t*_(38.1)_ = 5.59, *p* < 0.001; Monophonic EI: *M* = 38.25, CIs = 32.57, 43.94; Monophonic EN: *M* = 40.47, CIs = 33.58, 47.36; Monophonic PI: *M* = 38.97, CIs = 33.94, 44.00; Monophonic RE: *M* = 46.92, CIs = 42.36, 51.48]. In the Stereo Presentation mode (Figure 1A), participants attributed significantly higher scores on Realism than on Emotional Involvement [*t*_(33.8)_ = 6.13, *p* < 0.001] and Enjoyment [*t*_(32.6)_ = 4.28, *p* < 0.001; Stereo EI: *M* = 46.66, CIs = 41.93, 51.39; Stereo EN: *M* = 47.12, CIs = 40.9, 53.28; Stereo PI: *M* = 53.89, CIs = 50.02, 57.76; Stereo RE: *M* = 57.91, CIs = 54.71, 61.11]. In addition, participants attributed significantly higher scores to Physical Immersion than to Emotional Involvement [*t*_(39.4)_ = 6.43, *p* < 0.001]. In the Surround Presentation mode (Figure 1A), participants attributed significantly higher scores on Realism than on Emotional Involvement [*t*_(34.4)_ = 5.86, *p* < 0.001] and Enjoyment [*t*_(32.7)_ = 3.93, *p* < 0.001; Surround EI: *M* = 54.09, CIs = 48.96, 59.22; Surround EN: *M* = 54.99, CIs = 48.53, 61.44; Surround PI: *M* = 63.78, CIs = 59.41, 68.15; Surround RE: *M* = 64.90, CIs = 61.10, 68.70]. Furthermore, participants attributed significantly higher scores to Physical Immersion than to Emotional Involvement [*t*_(41.4)_ =8.5, *p* < 0.001) and Enjoyment [*t*_(33.3)_ = 4.02, *p* < 0.001]. Moreover, in all questions (Figure 1B) participants always attributed significantly higher absolute scores when stimuli were presented in the Surround Presentation mode than when they were presented in the Stereo Presentation mode [EI: *t*_(42.1)_ = 6.64, *p* < 0.001; EN: *t*_(41.9)_ = 7.03, *p* < 0.001; PI: *t*_(42.7)_ = 8.81, *p* < 0.001; RE: *t*_(42.5)_ = 6.23, *p* < 0.001] or in the Monophonic Presentation mode [EI: *t*_(33.18)_ = 6.61, *p* < 0.001; EN: *t*_(32.9)_ = 6.07, *p* < 0.001; PI: *t*_(33.5)_ = 10.33, *p* < 0.001; RE: *t*_(33.3)_ = 7.5, *p* < 0.001]. Moreover, independently from the question, participants attributed significantly higher scores when stimuli were presented in the Stereo Presentation mode than when they were presented in the Monophonic Presentation mode [EI: *t*_(35.2)_ = 4.97, *p* < 0.001; EN: *t*_(35)_ = 3.94, *p* < 0.001; PI: *t*_(35.8)_ = 8.8, *p* < 0.001; RE: *t*_(35.6)_ = 6.49, *p* < 0.001].

### Discussion

In this first behavioral experiment, we used a diverse set of naturalistic stimuli consisting of validated cinematic audio excerpts. This approach allowed for a more diverse range of stimuli and more generalizable results (Sonkusare et al., 2019) compared to previous studies (Lipscomb and Kerins, 2004). We investigated how different audio Presentation modes affect the emotional and bodily involvement and audio perception of participants. Results showed that participants consistently gave higher ratings when stimuli were presented in the Surround Presentation mode compared to the Monophonic or Stereo Presentation modes. Specifically, we found that the Surround Presentation mode was particularly effective in eliciting a sense of Realism, Emotional Involvement, and Physical Immersion among participants. These data are in line with the meta-analysis by Cummings and Bailenson, who reported that the spatial presence experience, evoked by the Surround Presentation mode, correlates positively with the level of immersion of the system (Cummings and Bailenson, 2015). We also corroborate, with more robust results and heterogeneous and ecological stimuli, previous results confirming that the sense of presence can be heightened by the spatialized sound Presentation mode (Lessiter and Freeman, 2001; Västfjäll, 2003; Kobayashi et al., 2015).

## Experiment 2

### Materials and methods

#### Participants

Twenty-four participants (*N* = 24, 11 men and 13 women, with a mean age *M* of 24.3 years and standard deviation SD of ±2.4, within a range of 21 to 30 years) were selected using an adaptation of the generalized listener selection (GLS) procedure (Mattila and Zacharov, 2001; Bech and Zacharov, 2006). For questionnaire descriptive statistics, see Supplementary material. Participants had a high education level (*M* = 15.2 years, SD = ± 1.5 years), had no prior history of neurological or psychiatric disorders, were right-handed as determined by the Italian version of the Edinburgh Handedness Inventory (Oldfield, 1971), had discriminative abilities of both loudness and sound source localization, had normal hearing acuity, and were “un-trained/naive subjects” as described in ITU-T Recommendation P.800 (ITU-R, 1996). All participants provided written informed consent to participate in the studies, which were approved by the local ethical committee “Comitato Etico Area Vasta Emilia Nord” and were conducted in accordance with the 1964 Declaration of Helsinki and its later amendments or comparable ethical standards (World Medical Association, 2013).

#### Stimuli

We used the same set of 27 cinematic excerpts without music and dialogues used in Experiment 1. A set of 27 control stimuli were also generated by phase-scrambling the original audio excerpts making them unintelligible to the participant but retaining all the acoustic characteristics on the frequency level and the same duration (10 s). Based on the results of the first experiment and for the purpose of simplifying the experimental paradigm, we chose the most and least effective among the three Presentation modes, Surround and Monophonic, for a total of 108 experimental stimuli.

#### Procedure

Participants listened to 27 cinematic excerpts and 27 control excerpts reproduced in two Presentation modes, played randomly twice for a total of 216 trials. The experiment was divided into four blocks of 54 trials each, with a break between blocks for a total experiment duration of ~60 m. Each trial consisted of a black fixation cross on a gray background (1.5 s), followed by the auditory stimulus presented for 10 s on a black screen. After viewing the stimulus, participants had 5 s to respond to a question on a Visual Analog Scale (VAS) from 0 to 100: “How much did you feel physically immersed?” (Physical Immersion, PI). We used only one question compared to Experiment 1 in order to reduce the complexity of the task and we chose one of the questions that better characterizes the spatialized sound experience as exposed in Experiment 1. Before the experiment, we trained participants with six trials, three per each Presentation mode, using stimuli previously excluded through the validation process. A gray background was used as an inter-trial interval (ITI) with a duration of 3.5 s. After the experimental task, participants were asked to indicate if they recognized any action-related sound. If they stated that there was an action-related sound present, they were asked to write down what action sound they recognized. This information was used to verify that participants were able, on average, to recognize one action-related sound in each stimulus. At the end of the experimental session, the participant was asked to fill out the Film Immersive Experience Questionnaire (F-IEQ) (Rigby et al., 2019). For questionnaire descriptives, see Supplementary material. Stimuli were presented with MATLAB extension Psychtoolbox-3 (Brainard, 1997).

#### EEG and EMG recording and pre-processing

The electromyography (EMG) signal was acquired by an AD Instruments PowerLab 35 (ADInstruments, U.K.), and LabChart 8 Pro software was used for recording. EMG activity was bipolarly recorded on the left Extensor Digitorum Communis and left tibialis anterior with 4 mm standard Ag/Ag-Cl electrodes. Before being attached to the muscle regions, the participants' skin was cleaned with an alcohol solution and the electrodes were filled with gel electrode paste (Fridlund and Cacioppo, 1986). EMG was sampled at 2 kHz and recorded with an online Mains Filter (adaptive 50 Hz filter). A band-pass filter (20–500 Hz) was applied, and data were arithmetically rectified (Abs). We calculated the EMG root-mean-square (RMS) response in microvolts (μV) by subtracting the baseline activity (average activity during fixation cross) from the activity during each stimulus divided into 20 segments of 500 ms each. EMG recording was done to exclude that the desynchronization recorded during stimulus presentation was influenced by participants' movements. Hence, outliers (segments with EMG activity ±3 SDs from baseline RMS) were considered movement artifacts, leading to trial exclusion during EEG pre-processing.

EEG data were acquired by a Geodesic Sensor System which includes the Net Amps 300 amplifier and a 128-channel HydroCel Geodesic Sensor Net (HCGSN-128) and recorded at a sampling rate of 500 Hz with the vertex (Cz) as an online reference while sensor-skin impedances were maintained below 50 kΩ for each sensor using Net Station 5.4 EGI software (Electrical Geodesic Inc., Eugene, OR). We applied a high-pass filter (0.5 Hz, transition window of 0.25 Hz) and ZapLine line noise removal (50 Hz Notch) using MATLAB (MathWorks, 2022) toolbox EEGLAB v2022.1 (Delorme and Makeig, 2004). Bad channels were identified with *Clean Rawdata* EEGLAB plug-in (v2.0) using flatline criterion (max 5s), line noise criterion (cutoff SD = 4), and minimum channel correlation (cutoff *r* = 0.7) and were interpolated using the spherical interpolation method. We removed 24 channels that were located at the periphery or the frontal region of the sensor net as they were likely to show residual muscle (13 peripheral channels: Ch48, Ch49, Ch56, Ch63, Ch68, Ch73, Ch81, Ch88, Ch94, Ch99, Ch107, Ch113, and Ch119) and eye artifacts (11 frontal channels: Ch1, Ch8, Ch14, Ch17, Ch21, Ch25, Ch32, Ch125, Ch126, Ch127, and Ch128), reducing the number of channels from 128 to 104. Continuous EEG data were divided into 12 s epochs, which included 2 s of baseline and 10 s of activity during the presentation of the stimulus. Epochs with muscle activity, identified using EMG (see above), were removed. Independent component analysis (ICA) was applied, and an automated recognition algorithm (MARA) was used to identify ocular, cardiac, and muscular artifacts (Winkler et al., 2011, 2014). A mean number of 16.7 (SD = 1.8) independent components (ICs) were removed. Finally, EEG data were re-referenced to the common average (Bertrand et al., 1985).

#### Analysis

In order to investigate the dynamic changes in spectral power over time and temporal patterns of neural activity related to the perception of audio Presentation modes, we performed a time-frequency analysis using the Hanning taper method. The window length was fixed at 0.5 s, with a frequency resolution of 1 Hz, spanning from 3 to 32 Hz. This allowed for the examination of event-related spectral perturbation (ERSP) in Alpha (8–13 Hz) and Beta (14–32 Hz) frequency bands. Data were baseline corrected by division considering as a baseline the 500 ms window before the stimulus onset. We averaged the Monophonic Control Presentation mode and the Surround Control Presentation mode with the aim to obtain a control condition independent from the perceptual differences due to Presentation modes and considered hereinafter as a Control Presentation mode.

In order to address the multiple comparisons problem (MCP) that arises from the multidimensional EEG data structure and to control for family-wise error rate (FWER), we used a cluster-based non-parametric test for within-subjects experiments implemented in FieldTrip (Oostenveld et al., 2011). The cluster-based test statistics is calculated by comparing experimental conditions at the sample level, selecting samples with *t*-values above a certain threshold, clustering them based on temporal, spatial, and spectral adjacency, and taking the sum of *t*-values within each cluster. The significance probability is then calculated using the Monte Carlo permutation method with 500 random draws. A *p*-value is calculated by comparing the observed test statistic to the distribution of test statistics obtained through random partitions of the data. A cluster is considered significant if its *p*-value is less than the critical Alpha level of 0.05. This data-driven approach allows one to identify specific time windows and electrode clusters, where there is a significant difference in neural activity between experimental conditions without any spatial cluster and frequency band assumption, and highlight regions of interest for further analysis. From electrodes in the identified clusters, we then extracted the log-ratio frequency power within the significant time window/frequencies range, and a linear mixed effect analysis was performed. Following a hierarchical approach, we initially created a simple model using one parameter, and we progressively added others with the aim to evaluate whether their inclusion improved model fit. Likelihood ratio tests, Akaike Information Criterion (AIC), and Bayesian Information Criterion (BIC) were used to rigorously choose which parameters improved model fit. We entered log-ratio frequency power as the dependent variable and Presentation modes (three levels: Surround, Monophonic, and Control) as independent fixed variables. The participants were included as a random intercept. This approach accounted for the within-subject and between-subject variability in the data. Outliers were identified and excluded from the analysis based on the standardized model residuals and a threshold value of Cook's distance (threshold = 1). *Post-hoc* tests were conducted using Tukey's correction for multiple comparisons and Kenward–Roger degrees-of-freedom approximation method.

For behavioral analysis and its results, see Supplementary material. Statistical analyses were performed using R software (R Core Team, 2022), lme4 (Bates et al., 2015), effects (Fox and Weisberg, 2019), and emmeans (Lenth, 2022) packages. For data plotting, we used the ggplot2 (Wickham, 2016) package.

### Results

#### Alpha band range

**Figure 2:**
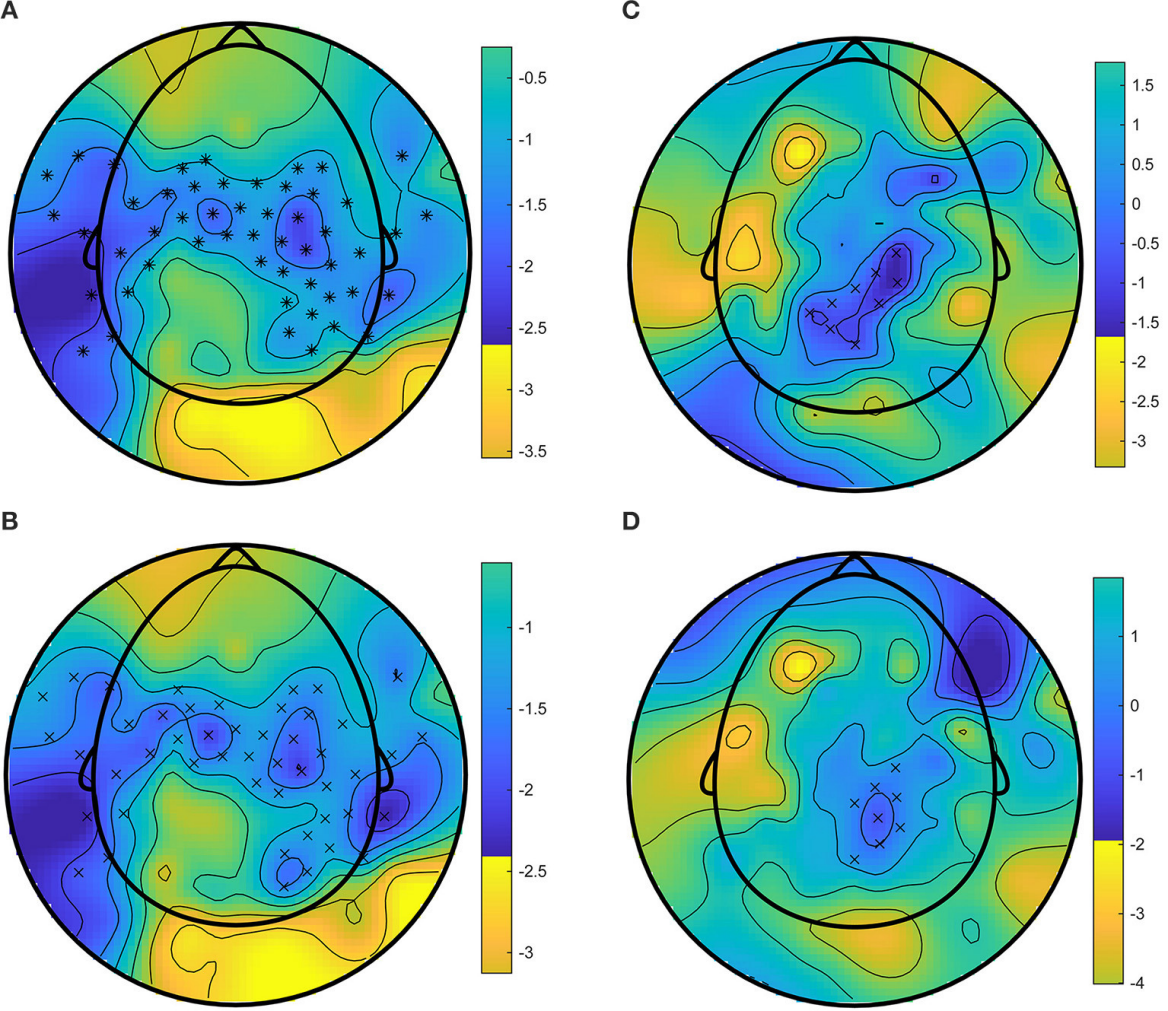
**(A)** Surround—Monophonic Alpha band (8–10 Hz) cluster (peak time 5 s from stimulus onset). **(B)** Surround—Control Alpha band (8–10 Hz) cluster (peak time 5 s from stimulus onset). **(C)** Surround—Monophonic Low-Beta band (16–18 Hz) cluster (peak time 4.5 s from the stimulus onset). **(D)** Surround—Control Low-Beta band (16–18 Hz) cluster (peak time 4.5 s from the stimulus onset). The Crosses (+) symbol indicates channels with *p* < 0.01 and asterisks (^*^) indicates channels with *p* < 0.001.

A significant cluster in central and parietal areas (see Supplementary Figure S3 for cluster channels) was identified in the time window from 3 to 7 s in the Alpha frequency band (8–10 Hz). This reflects a difference in neural activity in this frequency band and time window between the experimental conditions. Specifically, this cluster is characterized by a significantly higher event-related desynchronization (ERD) during the Surround Presentation mode compared to both the Monophonic (Figure 2A) and Control Presentation modes (Figure 2B), with a peak difference of ~5 s from the stimulus onset.

**Figure 3:**
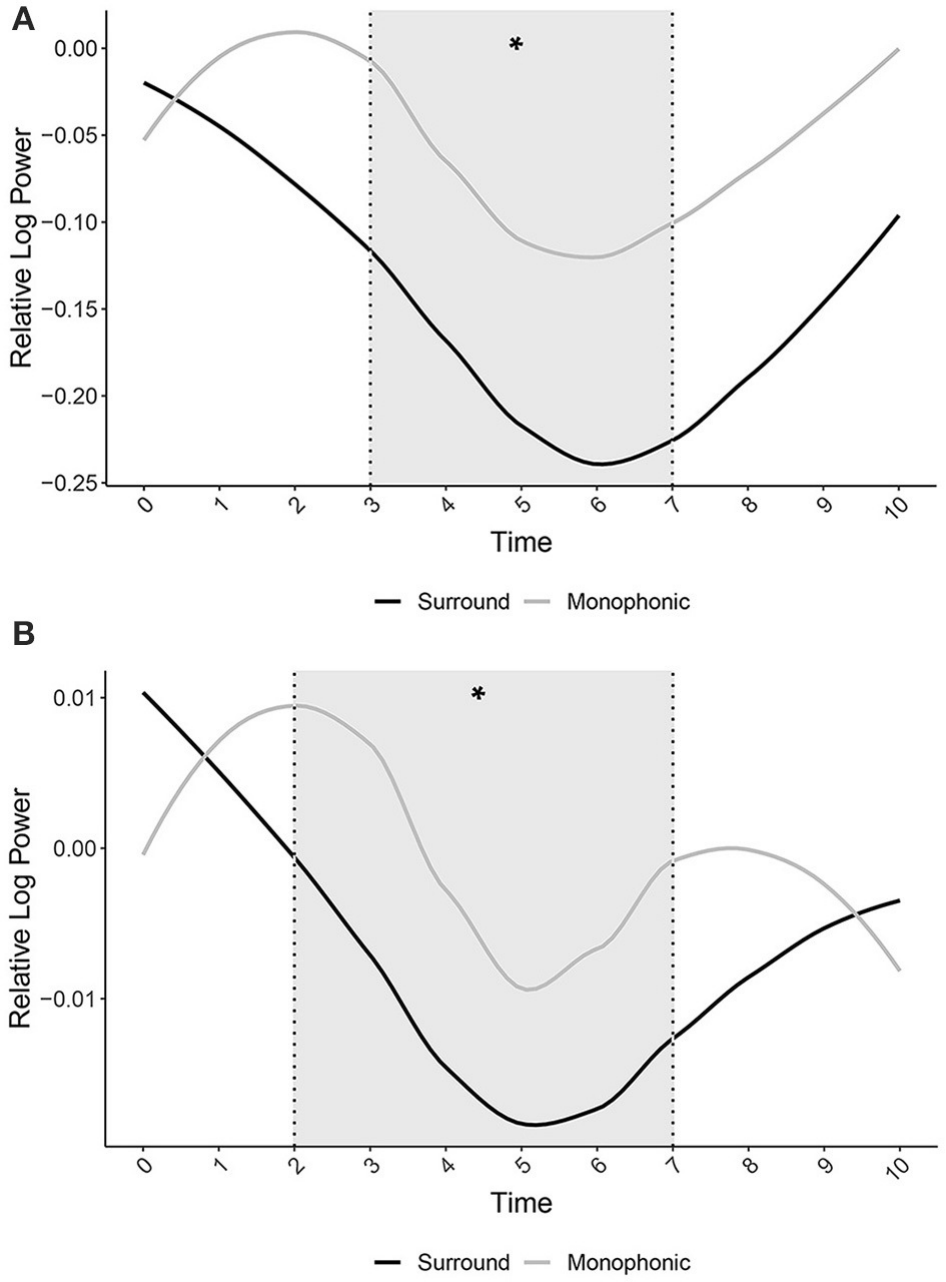
**(A)** Log-ratio frequency power extracted from the Surround Presentation mode and the Monophonic Presentation mode relative to the Control in the Alpha cluster over time. **(B)** Log-ratio frequency power extracted from the Surround Presentation mode and the Monophonic Presentation mode relative to the Control in the Low-Beta cluster over time. Gray area highlights indicate significant differences between Presentation modes, asterisk (^*^) = *p* < 0.05.

The linear mixed model on log-ratio frequency power explained 57% of the variance in a dependent variable taking into account the random effects ($R_{\text{m}}^{2}$ = 0.11; $R_{\text{c}}^{2}$ = 0.57). The model revealed a significant main effect of Presentation modes [$\chi_{\left(2\right)}^{2}$ = 43.87, *p* < 0.001], showing that there was a significantly greater ERD in the Surround Presentation mode when compared to both the Monophonic [*t*_(16, 777)_ = 3.79, *p* < 0.001] and Control Presentation modes [*t*_(16, 777)_ = 6.62, *p* < 0.001; Surround: *M* = 1.5, CIs = 0.83, 2.17; Monophonic: *M* = 1.6, CIs = 0.93, 2.28; Control: *M* = 1.66, CIs = 0.98, 2.33]. This means that there was an increase in neural activity in the centro-parietal areas during the Surround Presentation mode when compared to the Monophonic and Control Presentation modes, with a peak difference of ~5 s after the stimulus onset. Furthermore, the *post-hoc* comparisons also showed that there was a significantly greater ERD in the Monophonic Presentation mode when compared to the Control Presentation mode [*t*_(16, 777)_ = 2.25, *p* < 0.001]. In order to better visualize the time course and patterns of ERD, we normalized Surround and Monophonic Presentation mode power by subtracting Control Presentation mode power. We detected a different ERD pattern between the Surround and Monophonic Presentation modes (Figure 3A). Even if a significant difference is detected in the time windows between 3 and 7 s after the stimulus onset, the ERD in Surround starts after the stimulus onset followed by a power rebound 6 s after the stimulus onset, while in Monophonic, we distinguish an ERS in the first 2 s after the stimulus onset and an ERD 2 s after the stimulus onset followed by a power rebound 6 s after the stimulus onset (Figure 3A). The findings demonstrated that the temporal progression of cortical activation differed between the two Presentation modes.

#### Low Beta band range

The second significant cluster in the central area (see Supplementary Figure S4 for cluster channels) was identified in the time window from 2 to 7 s in the Low-Beta frequency band (16–18 Hz). This reflects a difference in neural activity in this frequency band and time window between the experimental conditions. Similar to the first Alpha cluster, this cluster is also characterized by an ERD during the Surround Presentation mode compared to both the Monophonic (Figure 2C) and Control Presentation modes (Figure 2D), with a peak difference of ~4.5 s from the stimulus onset.

The linear mixed model on log-ratio frequency power explained 89% of the variance in a dependent variable taking into account the random effects ($R_{\text{m}}^{2}$ = 0.2; $R_{\text{c}}^{2}$ = 0.89). The model revealed a significant main effect of Presentation modes [$\chi_{\left(2\right)}^{2}$ = 9.79, *p* < 0.001], showing that there was a significant ERD in the Surround Presentation mode when compared to both Monophonic [*t*_(5017)_ = 2.58, *p* < 0.05] and Control Presentation modes [*t*_(5017)_ = 2.94, *p* < 0.001; Surround: *M* = 0.29, CIs = 0.17, 0.40; Monophonic: *M* = 0.31, CIs = 0.18, 0.41; Control: *M* = 0.30, CIs = 0.18, 0.41]. This means that there was an increase in neural activity in the centro-parietal areas during the Surround Presentation mode when compared to the Monophonic and Control Presentation modes with a peak difference of ~4.5 s after the stimulus onset. In order to better visualize the time course and patterns of ERD, we normalized Surround and Monophonic Presentation mode power by subtracting Control Presentation mode power. Moreover, in this cluster, we detect a partially different ERD pattern between the Surround and Monophonic Presentation modes (Figure 3B). Even if the significant difference is detected 2 s after the stimulus onset, the ERD in the Surround starts after the stimulus onset followed by a power rebound 6 s after the stimulus onset, while in Monophonic, we distinguish an ERS in the first 2 s after the stimulus onset and an ERD 2 s after the stimulus onset followed by a power rebound 5 s after the stimulus onset.

### Discussion

The objective of this HD-EEG experiment was to explore the neural cortical mechanisms and the temporal specificity of sound perception when presented in two distinct acoustic Presentation modes, namely, Monophonic and Surround. The main focus was to compare the neural activity in the Surround Presentation mode to that in the Monophonic and Control Presentation modes, with the hypothesis that the enhanced spatialization of sound in the Surround Presentation mode would lead to greater activation of embodied simulation mechanisms viewed as the physiological index of the sense of presence. Using a data-driven approach that allowed us to identify specific time windows and electrode clusters where there is a significant difference in neural activity between experimental conditions without any spatial cluster and frequency band assumption, we identified two significant centro-parietal clusters: the first in the Alpha frequency band (8 to 10 Hz) and in the time window from 3 to 7 s and the second in the Low-Beta frequency band (16 to 18 Hz) and in the time window from 2 to 7 s. Since the Rolandic Alpha frequency band of interest (8–13 Hz) overlaps with the occipital Alpha band, recordings in central areas might be affected by this posterior activity. However, given that significant clusters were detected only in central and parietal areas, we can exclude that our results were related to attentional/vigilance factors originating from the parieto-occipital cortex. Further analysis revealed a significant ERD in the Surround Presentation mode when compared to both Monophonic and Control Presentation modes both in Alpha and Low-Beta centro-parietal clusters, confirming previous results (Tsuchida et al., 2015) using a more robust analysis approach. We observed a late significant ERD peak (~4.5/5 s) compared with the typical time course of mu rhythm desynchronization (Avanzini et al., 2012).

## General discussion

The results of the present study provide novel insights into the relationship between virtual acoustic spatial environments and the sense of presence providing evidence that Surround Presentation mode enhances the sense of presence by activating embodied simulation mechanisms. In Experiment 1 and consistently with previous research on the relationship between Surround sound and the cinematic experience (Lessiter and Freeman, 2001; Västfjäll, 2003; Pettey et al., 2010; Kobayashi et al., 2015), participants explicitly reported that the Surround Presentation mode significantly enhances the sense of presence, Emotional Involvement, and Physical Immersion, thus showing that the Surround Presentation mode enhances immersion by more closely approximating real-world auditory experience. These findings are consistent with previous research showing that Surround sound formats can envelop the viewer in a 360-degree auditory space unlike the 180-degree space of stereo or mono sound (DiDonato, 2010). Cummings et al., using a meta-analytic approach, investigated the relationship between the immersive quality of a mediated environment and the level of presence experienced by the participant. Several immersive features that offer high-fidelity simulations of reality such as Surround sound had a significant effect on presence (Cummings and Bailenson, 2015). Additionally, these results offer some interesting theoretical implications, supporting the formation of presence as outlined by the spatial situational model framework proposed by Wirth et al. (2007).

The level of similarity between the perceptual experience elicited by video clips and the visual experience during real-life movements is believed to depend on the filming technique. The results of Heimann et al. indeed suggest that there may be a relationship between the perception of approaching stimuli and the feeling of involvement in the scene (Heimann et al., 2014, 2019). This may be due to the presence of more depth cues, which more closely resemble real-life vision. A similar mechanism can be hypothesized for the audio component in cinematic immersion, where the Surround Presentation mode can more closely resemble real-life hearing and activate embodied simulation processes. The EEG results of Experiment 2 further support this hypothesis. The Surround Presentation mode elicited a higher peak of Alpha rhythm desynchronization, reflecting greater activation of the mirror mechanism which represents the neural basis of embodied simulation. The desynchronization peak was delayed from the onset of stimulus presentation likely because of the naturalistic and, to some degree, heterogeneous stimuli used, which lacked a clearly time-locked goal-oriented action onset. This may have influenced the timing of the neural response observed in our study as the sound of the actions that were present in the stimuli had a high and diverse temporal dynamicity. Overall, these findings highlight the importance of considering the nature and characteristics of stimuli used in experiments, particularly when investigating the temporal specificity of neural responses. Furthermore, it is also possible that the use of dynamic and naturalistic stimuli, as opposed to experimental stimuli created *ad hoc*, may have led to a more complex and nuanced neural response. Our findings are consistent with previous studies that have reported different EEG topographies for the Alpha and Beta components of the mu rhythm (Pfurtscheller et al., 1994; McFarland et al., 2000). Previous research revealed different source locations and reactivity for the Alpha and Beta subcomponents of the mu rhythm desynchronization active during action execution and action observation, supporting the idea that they serve distinct functions (Hari and Salmelin, 1997; Pfurtscheller et al., 1997; Hari, 2006; Press et al., 2011; Hobson and Bishop, 2016). The Alpha subcomponent is thought to reflect a sensorimotor function, while the Beta component is more closely linked to motor cortical control. Indeed, further research is needed to fully understand the underlying mechanisms and factors that contribute to this neural response, the different functions of Alpha and Beta subcomponents and how they arise from different neural networks, and the functional significance of the activation of embodied simulation mechanisms in acoustic cinematic immersion. Regardless of these future developments, we can state that immersion, as an objective property of the playback system, a defining characteristic of our stimuli delivery setup, was reflected by the instauration of the sense of presence revealed by a stronger embodiment of spectators. These findings support the idea that cinematic experience is unique and directly connected to sensory-motor patterns that connect the viewer with the screen, allowing for a form of immersive simulation that exploits the full potential of our brain-body system (Freedberg and Gallese, 2007; Gallese and Guerra, 2012, 2019, 2022; Fingerhut and Heimann, 2022). The result is an intersubjective relationship between the viewer and the film that blurs the boundary between the real and imaginary worlds (Gallese and Guerra, 2019).

## Conclusion

This study provides new data on how increasing the spatial complexity of virtual environments mediated by cinematic sequences can increase the participant's sense of presence. The greater neural activity recorded in the centro-parietal areas can contribute to the understanding of the neural mechanisms of embodied spatialized auditory perception. In future, by further understanding how the integration between sound and visual information in the cinematic experience occurs, we can gain insight into how the brain processes this information and how it can be used to enhance the viewers' immersive experience. Furthermore, deeper comprehension can also be applied to other areas such as virtual reality and augmented reality, which also rely on the integration of sound and visual information to create immersive experiences. Filmmakers and sound designers may also leverage this knowledge to precisely manipulate audiovisual elements, resulting in a heightened emotional impact and greater engagement with film scenes. The knowledge gained from this exploration should also have broader implications in fields beyond entertainment. Fields such as education and therapy can benefit from a deeper insight into how the brain processes and integrates sound and visual information. These applications can range from designing effective educational multimedia content to developing immersive training for therapy/ rehabilitation purposes.

## Data availability statement

The datasets presented in this study can be found in online repositories. The names of the repository/repositories and accession number(s) can be found at: https://osf.io/ntemf/?view_only=a6f1d81e84d940f9a31513b9ed3f8c67.

## Ethics statement

The studies involving human participants were reviewed and approved by “Comitato Etico Area Vasta Emilia Nord” and were conducted in accordance with the 1964 Declaration of Helsinki and its later amendments or comparable ethical standards (World Medical Association, 2013). Participants provided their written informed consent to participate in these studies.

## Author contributions

NL and VS conceptualized the idea and discussed it with all the authors. NL edited the stimuli, performed data acquisition, performed analyses, and wrote the manuscript. All authors designed the experiment, interpreted the results, contributed, and approved the manuscript.
