# Supplementary material for: The effect of Surround sound on embodiment and sense of presence in cinematic experience: a behavioral and HD-EEG study
Source: Front Neurosci. 2023 Sep 7;17:1222472. doi: 10.3389/fnins.2023.1222472 (PMC10513788; doi:10.3389/fnins.2023.1222472)
Supplement: Supplementary file 1 [file Data_Sheet_1.docx]

**Supplementary Materials**

**The effect of Surround sound on embodied simulation and sense of Presence in cinematic experience: an HD-EEG study**

Nunzio Langiulli^1^, Marta Calbi^2^, Valerio Sbravatti^3^, Maria Alessandra Umiltà^4,5^, Vittorio Gallese^1,5^

^1^ University of Parma, Department of Medicine and Surgery, Unit of Neuroscience, Parma, Italy

^2^ State University of Milan, Department of Philosophy “Piero Martinetti”, Milan, Italy

^3^ Sapienza University of Rome, Department of History, Anthropology, Religions, Arts and Performing Arts, Rome, Italy

^4^ University of Parma, Department of Food and Drug, Parma, Italy

^5^Italian Academy for Advanced Studies in America at Columbia University, New York, USA

*Corresponding author

University of Parma, Department of Medicine and Surgery, Unit of Neuroscience, Parma, Italy E-mail: [nunzio.langiulli@unipr.it](mailto:nunzio.langiulli@unipr.it)

**Stimuli Validation Experiment**

**Materials & Methods**

**Participants**

One hundred participants (67 males and 33 females, with mean age M of 23.6 and standard deviation SD of ± 4.9 years, within a range of 18 to 38 years) took part in the Stimuli Validation Experiment. Recruitment took place online through the Prolific platform (Palan & Schitter, 2018). All participants were fluent in English and had no prior history of neurological or psychiatric disorders. All participants were rewarded through the Prolific platform with an hourly rate of approximately £7.5/hour. All participants provided written informed consent to participate in the studies, which were approved by the local ethical committee “Comitato Etico Area Vasta Emilia Nord” and were conducted in accordance with the 1964 Declaration of Helsinki and its later amendments or comparable ethical standards (World Medical Association, 2013).

**Stimuli**

We extracted a set of 185 cinematic excerpts (10 s long) from Academy Award-nominated feature films in the categories of "Best Sound" and "Best Sound Editing" from the editions between 1979 and 2019. Stimuli were chosen based on three criteria: (1) no music must be present to ensure that the emotional response elicited by the excerpt is not influenced by the presence of music, which can often be used to enhance emotional responses (Wöllner et al., 2018); (2) there must be no dialogue that can often give context or emotional information, which can activate cognitive processes related to human voice perception that are not relevant to the experiment (Latinus & Belin, 2011); (3) the excerpt must be characterized by a dynamic acoustic spatialization with different “sound objects” realistically moving in the sound space.

The original video in Advanced Video Codec (AVC) format and the Stereo (2-channels) audio in Audio Coding 3 (AC-3) format from the MatrosKa audio-video container were converted in MPEG-4 Part 14 (ISO/IEC 14496-14:2003) audio-video container format for compatibility reasons. The audio track was converted in Advanced Audio Codec Low Complexity (AAC LL) with no modifications to the audio bitrate and sampling frequency.

**Procedure**

Participants were randomly divided into 10 groups of 10 participants each. From the set of 185 stimuli, 19 stimuli were randomly assigned to nine groups and 14 stimuli to one group (pilot group). Stimuli were repeated three times for a total of 57 trials (42 for the control group) and for a total duration of about 15 m. A pilot group was utilized in order to assess the experimental paradigm, to ensure that the procedure was being executed as intended and to identify any potential issues with the methodology. Participants were instructed to listen/watch to randomly presented stimuli, exclusively on a PC, using headphones or external speakers. Each stimulus was repeated three times and was followed by one of three randomly selected questions: (1) “How would you judge the Dynamicity of the scene?”, (2) “How would you judge the Emotional Valence of the scene?”, (3) “How would you judge the Emotional Intensity of the scene?” (All questions are translated from Italian). Hence, the questions required the participant to make a personal judgment about the Dynamism, Emotional Valence, and Emotional Intensity of audio-visual stimuli using a Visual Analogue Scale (VAS) ranging from 0 to 100 for the questions on Dynamism and Emotional Intensity, and from -50 to +50 for the question on Emotional Valence. In addition to VAS scores, response times (RTs) were recorded.

The experimental paradigm for stimulus validation was coded in PsychoPy (v3.0) and hosted by the Pavlovia service for *online* program execution and management of collected data (Peirce et al., 2019).

**Analysis & Stimuli Selection**

In order to exclude potential participants’ errors due to accidental mouse clicks or distractions during the online execution (Sauter et al., 2020), scores assigned with response times (RTs) less than or equal to 500 ms or greater than 10 s were excluded from the analysis. This resulted in the exclusion of 303 data points from the analysis. Outlier detection was also performed on the scores assigned to each stimulus for each question. This resulted in the exclusion of additional 183 data points from the analysis.

There is evidence to suggest a correlation between immersion and intensity of perceived emotions as found by Visch and colleagues (Visch et al., 2010). Additionally, negative emotions have been shown to have processing priority, triggering defense mechanisms through early activation of the amygdala (Figueiredo et al., 2003; Leppänen & Nelson, 2009). Furthermore, research has shown that audio-visual stimuli with negative valence extracted from feature films can induce greater arousal than stimuli with positive valence (Fernández-Aguilar et al., 2019). With these findings in mind, stimuli were chosen based on three criteria (see Figure S1): (1) Dynamism score higher than the sample Dynamism median score (*Md* Dynamism = 66), (2) Emotional Intensity score higher than the sample Emotional Intensity median score (*Md E*motional Intensity = 66), (3) Emotional Valence score lower than the sample Emotional Valence median score (*Md* Emotional Valence = -14).


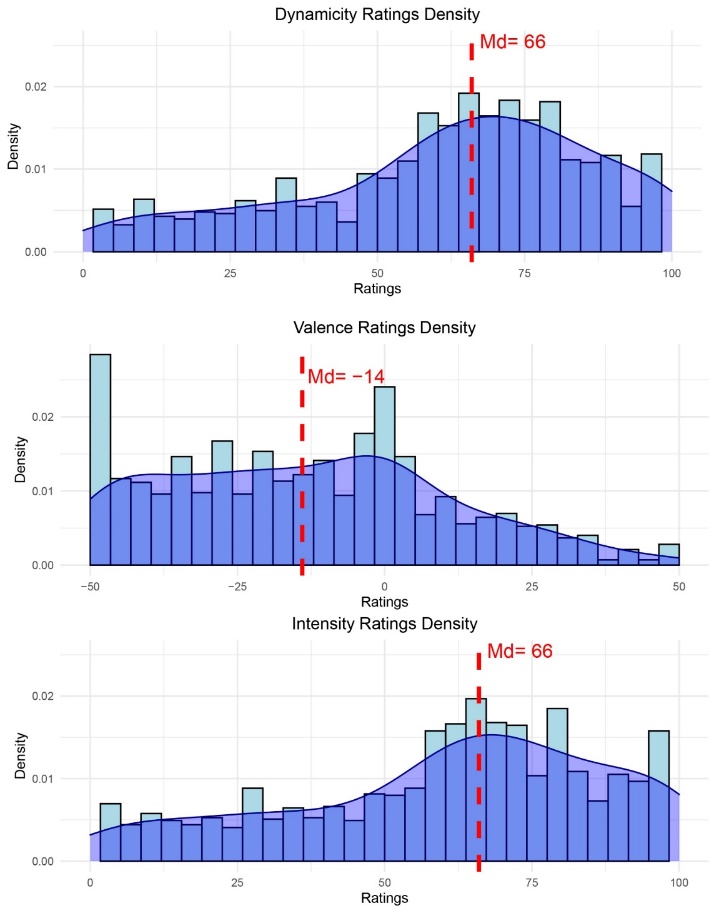


**Figure S1.** Scores Density Distributions and sample Medians (Md, dashed red line).

We selected 50 stimuli that had the desired characteristics, from the initial sample of 185 stimuli sample, with a mean Dynamicity score of 75, mean Valence score of -23 and mean Intensity score of 78 (Table S1).

|  | **Mean** | **SE** | **Min** | **Max** | **IQR** | **Range** |
| --- | --- | --- | --- | --- | --- | --- |
| Dynamicity | 74.95 | 0.83 | 67.2 | 94.7 | 9.1 | 27.6 |
| Emotional Valence | -23.21 | 0.84 | -40.4 | -14.4 | 8 | 26.1 |
| Emotional Intensity | 77.71 | 0.77 | 67.6 | 90.1 | 8.14 | 22.5 |

**Table S1**. Summary of Selected Stimuli Scores (SE= Standard Error, IQR=Interquartile Range).

**Acoustic Formats & Features Analysis**

For the selected 50 stimuli set, the original 5.1-channels Surround audio track encoded in Audio Coding 3 (AC-3) format from the MatrosKa audio-video container was converted in Pulse Code Modulation (PCM) format and saved in a Waveform Audio File container (sample rate= 48000 Hz; sample size= 16 bits). This original Surround sound version (5.1-channels) was composed by six audio channels (“L” = Left, “R” = Right, “C” = Center, “Ls” = Left Surround, “Rs” = Right Surround, “LFE” = Low Frequency Effects) and was used to derive a Stereophonic sound version (2-channels), composed by two audio channels (“L” = Left, “R” = Right), and a Monophonic sound version (1-channel) composed by one audio channels “C” = Center). The term Stereophonic refers to a sound reproduction paradigm that involves the use of two or more channels, with each channel carrying a different part of the audio signal. It's worth noting that even more advanced sound systems, such as 5.1 Surround sound, are technically still Stereophonic, as they use multiple channels to create a three-dimensional sound field. Hence, we will use the term Stereo hereinafter to refer specifically to the 2-channel sound version. The conversion was performed using MATLAB v.R2021a (MathWorks, 2021) by downmixing (amplitude averaging) the Surround sound to the Stereo sound, and then further downmixing the Stereo sound to the Monophonic sound. In Stereo sound version the “L” channel was composed by the amplitude average of “L”, “Ls”, “C”, and “LFE” channels from the Surround sound version and the “R” channel was composed by the amplitude average of “R”, “Rs”, “C”, and “LFE” channels from the Surround sound version. In Monophonic sound version the “C” channel was composed by the amplitude average of all channels from the Surround sound version.

In order to perceptually uniform the acoustic features of our stimuli we extracted pitch and loudness, two perceptual properties of sound that can be quantified, measured and normalized, from all stimuli in the three sound versions (Surround, Stereo and Monophonic). The pitch of the acoustic stimuli was extracted using the YIN algorithm, a widely used pitch detection method in speech and music analysis implemented in MATLAB v.R2021a (MathWorks, 2021). The extracted pitch values were then analyzed to identify the range of pitches present in the stimuli sample. The minimum and maximum pitch values were determined and outliers, defined as pitch values ±3 SDs from mean, were identified and excluded from the sample. We excluded 11 stimuli from the initial sample of 50 stimuli. The integrated loudness (overall average loudness) of the acoustic stimuli in The Loudness Unit Full Scale (LUFS) was extracted using MATLAB. In order to eliminate the potential influence of loudness level differences during the acoustic reproduction of stimuli we followed the European Broadcasting Union EBU R128 recommendation that defines the target loudness level at -23 LUFS (EBU, 2014). To normalize the stimuli to this level, a simple loudness adjustment operation was applied to the integrated loudness using MATLAB. In addition, the peak loudness and the loudness range were then analyzed. Peak loudness refers to the highest level of momentary loudness, which uses a sliding time window of 400 ms to describe instantaneous loudness, while loudness range is the difference between the highest and lowest level of momentary loudness within a sound or audio file. The minimum and maximum peak and range values were determined for all the stimuli. Outliers, defined as values values ±3 SDs from mean, were identified and excluded from the sample. We excluded 12 stimuli from the initial sample of 50 stimuli. Considering the exclusion of both pitch and loudness related outliers the final stimuli samples was composed by 27 stimuli.

**Stimuli Qualitative Descriptions**

In order to provide a detailed understanding of the acoustic properties of the stimuli selected we provided a description from a qualitative perspective, focusing on the different sound elements present in each stimulus and how they change over time with some emphasis on the different sound elements present in the surround channels (Ls and Rs) (Table S2). To clearly distinguish between the stimuli, each one was assigned a unique alphanumeric code.

| **Stimulus** | **Qualitative Description** |
| --- | --- |
| AS04 | Gunshots, slight clang. Acute impact of bullet on the driver’s body. Blood spurts on the glass. Machine gun fire. Wagon motor, explosion, wagon impact on another car and then on ruins. Soldiers’ groans in center channel. Wagon arrests. Ricochet. Metal sound of bullets hitting the ground. LFE regularly used. Impacts and gunshots prominent also in surround channels. |
| AS07 | Shots, ricochets, glass breaking. Metal sounds of bullets falling on the floor. Impact of the bullets on the wall. Point of audition clearly shifts consistently with the visual perspective. Surround channels are prominent (the left a bit more so), containing sounds of gunshots. LFE used slightly to enhance the gunshots. |
| BR02 | Moderately regular thuds of the head on the wall, groans. Noises of the wall breaking, fragments banging on the floor and impact of the two bodies falling. Most sounds are spread in all the five main channels. Point of audition shifts consistently with the point of view: full-frequency sounds in the room where the two men are, same sounds muffled heard through the wall in the adjacent room. LFE use subtly and then prominently, when the wall collapses and the two man fall. |
| BR09 | Mechanical noise of the missile approaching to the building. Explosions, glass breaking, debris impacts. The sound stream is slightly fragmented. Afterwards, the soundscape rarifies, there is far reverberation, while the point of view moves outside the building (long shot). Some subtle groans in center channel, spread in the surrounds which are regularly used – as is the LFE, albeit being prominent especially during the first explosions. |
| CA04 | Silent sound field, low background of the aircraft. Slight sound of the patch removed from the finger. Sudden, intense acoustic blast, reinforced by LFE and surround channels as well (which continue to be used though less prominently). Noisy sound field: air flux, impact of objects also in surrounds, metal and electromechanical sounds. |
| CA07 | Noises of impacts, electromechanical sounds, clatter. Hiss and air also from surround channels. LFE only at beginning and end. |
| CA11 | Spacious underwater point of audition, muffled. Water movements, hiss, string rubbing on metal, clink, creak. Ominous deep and cracking noises suggesting the movement of the aircraft. Sounds subtly spread in the surround channels. |
| CA13 | Rain, which spreads also in the surround. Crescendo of the turbine whir, explosion (includes LFE) accompanied by a couple of whistles. Right surround contains a very subtle whoosh (water or wind). |
| CA14 | Much muffled underwater sounds. Point of audition moves repeatedly under and above the water level. Sounds of water and rain (mixed also in the surround channels), wind, low animal-like sound of the airplane, distant electronic tones. |
| CA15 | Wind, rain, water moving and gurgling. Left surround contains mostly wind, right surround rain only. |
| HR02 | Shouts, moans, spread throughout the front channels (and for an instant in the surround ones as well). Gunshots, hisses, explosions, bloody impacts of bullets on bodies, footsteps running on the ground. Surrounds and LFE regularly used. |
| HR03 | Shouts, gunshots, bloody impacts of bullets on bodies, body impacts on the ground, hisses and explosions, hand grenade unlocked and thrown. Surrounds and LFE regularly used. |
| HR05 | Groans, mostly from the center channel but slightly spread throughout the other main channels. Gunshots, hisses and explosions, machine gun fire, bullet impact on the ground and on a soldier’s metal helmet. Surrounds and LFE regularly used. Two whooshes before the first bomb explosion are prominent in the right surround, consistently with the visual perspective. |
| HR09 | Hisses and explosions, shouts and groans (mostly from the center channel), gunshots, Wilhelm scream, clang, impact on ground. Surrounds and LFE regularly used. |
| HR10 | Gunshots, prominent in left surround. Shouts and groans, mostly from the center channel. Hisses and explosions, gunshots, various impacts (bullets on bodies, bodies on other bodies and on the ground). Surrounds and LFE regularly used, especially some gunshots from the left surround. |
| HR11 | Shouts and groans, mostly from the front channels. Gunshots, body impacts, hisses and explosions, mold falling over the soldiers, blood spurts. Surrounds and LFE regularly used. |
| HR15 | Cannon shots (much reinforced by LFE), explosions, distant shouts (spread throughout the five main channels), nearby breath (center channel only). Point of audition changes in terms of distance. Surrounds and LFE regularly used. |
| ID01 | Weapon release, explosion, glass shattering, screams (front channels, slightly spread in the surrounds), fire. Subtle growls. Surrounds and LFE regularly used. |
| ID05 | Roar of flames expanding towards the camera. Squeals, metal noises, explosions, glass shattering, screams, siren, animal-like voices and roars subtly mixed with the noises. Body impacts on car’s hood, breaking the windshield. Surrounds and LFE regularly used. |
| JU01 | Whir, impacts of the airplane on the branches and the trees, alarm, glass shattering, a woman’s scream, mechanical sounds, debris. Surrounds are prominent until the airplane stops, then they mute. LFE enhances most of the impacts. Various acoustic accents. |
| LS11 | Gunshots, explosions and debris in the center channel, bullet impacts. Bazooka launches missile which impacts on the ground. Human movements in the woods. Two musical chords (electronics and maybe low strings) in the background. Surrounds and LFE regularly used, the latter especially for the two explosions. |
| LS16 | Gunshots and ricochets, nearby groan in the center channel, distant shouts in the center channel but slightly spread throughout left and right. Much reverberation, also thanks to the surrounds which are regularly used. LFE enhances gunshots. |
| LS19 | Gunshots to the helicopter, shouts in the center channel, descending mechanical noises, blades whirring, thump of the helicopter breaking in two parts. Strong impacts of the falling helicopter on the mountain, some explosions. Surround (especially the left one) only at the beginning, then slightly for the first impact on the mountain, and in the end. Point of audition gets further consistently with the point of view. |
| MC04 | Noise of the cannon shot, series of impacts, wood clatter, roar of the ship, whooshes, sounds from the sea (objects sinking, ship sailing). Surrounds and LFE regularly used, the former being particularly prominent. |
| MC09 | Wind howling (almost choir-like), sea waves, squeak, ropes whooshes, roar of the ship, mast sinking in the sea. LFE used for an instant only, almost inaudible. Surrounds used constantly and prominently. |
| SK01 | Wheels on the rails, electricity buzzing, screeches, impacts, debris, glass. LFE used regularly and intensely. Surround channels are always used, especially during the first impact of the train and the one on the camera. The right one is slightly more prominent (along with the right front one), consistently with the visual perspective. |

**Table S2.** Stimuli Acoustic Qualitative Descriptions.

**Generalized Listener Selection Procedure**

Our adaptation of the Generalized Listener Selection (GLS) procedure described by Zacharov and colleagues (Bech & Zacharov, 2006; Mattila & Zacharov, 2001) included: six questionnaires, an audiometric test, two screening tasks about loudness discrimination and localization of the sound source. The screening tests were presented with MATLAB extension Psychtoolbox-3 (Brainard, 1997).

**Questionnaires**

Each participant had to fill out via Google Forms, before the experiment, a battery of questionnaires composed as follows:

1. GLS-1 Generalized Listener Selection (Bech & Zacharov, 2018);
2. QEAV (Questionnaire on Audio-Visual Experience);
3. B-MEQ Brief Music Experience Questionnaire (Werner et al., 2006);
4. IRI Interpersonal Reactivity Index (Albiero et al., 2006);
5. ITQ Immersive Tendencies Questionnaire (Witmer & Singer, 1998);
6. VMIQ-2 Vividness of Movement Imagery Questionnaire (Roberts et al., 2008).

Questionnaires were used to gather information about the participants' demographics, such as age, gender, education level, and cultural background, hearing history, knowledge of spatialization techniques, and movie-watching experience. These data were used to select a uniform sample of healthy "un-trained/naive subjects" as described in ITU-T Recommendation P.800 (ITU-R., 1996) to ensure that the participants did not have specific technical skills in the evaluation of acoustic reproduction systems.

**Audiometric Test**

Each participant was subjected, in the silent audiometric cabin, to a brief tonal audiometric test to measure hearing acuity threshold using the "Frequency Response of the Ear, Hearing Test" application developed in MATLAB (Rawashdeh, 2021). The participant wore *over-ear* headphones (Audio-Technica ATH-MSR7). A pure tone to both ears was simultaneously played, starting from a frequency of 1 KHz up to a frequency of 16 KHz with intervals of 1 KHz, at decreasing loudness levels. The participants’ task was to indicate whether he heard the played tone pressing a button. The next frequency in the series was played only when the participant failed to press the button, indicating that they were unable to hear the previous tone. The resulting audiometric curves were then compared with audiometric curves for otologically normal participants differentiated by age according to the ISO Standard 7029 (ISO, 2017) and participants whose hearing performance did not appear to be in the normal range were excluded from the experimental sample.

**Screening Task 1: Loudness Discrimination**

The first screening task was conducted to verify the participant's ability to discriminate sounds played at different loudness levels. The participant was positioned in the central listening position of the silent audiometric cabin. Five different acoustic stimuli, 500 ms long, were created and played simultaneously on five channels of the Surround sound system (LFE channel excluded). The stimuli consisted of pink noise sampled at 48000 Hz with different loudness levels. The highest loudness level was set at -23 LUFS, while the loudness level of other stimuli was set at 3 LUFS lower than the previous one, based on the minimum audible difference described by Larsen and colleagues (Larsen et al., 2008). The stimuli had the following loudness levels: -23 LUFS, -26 LUFS, -29 LUFS, -32 LUFS, -35 LUFS. The participant was given a forced-choice task, in which they had to listen to two stimuli played in succession and indicate which stimulus was played loudness level with the higher or whether both stimuli were played with the same loudness level. Stimuli were presented in all different possible pairings (including pairings between equal stimuli) for a total of 25 random trials. Each stimulus was accompanied by a visual cue, the first stimulus in the pair was accompanied by the label “First” while the second stimulus played was accompanied by the label “Second”. At the end of each playback, the question “Which sound had the loudest volume?” was displayed on the screen and the participant had to answer, within 5 s, with the mouse by choosing from the options “First”, “Equal” and “Second”. The cut-off criterion for participants’ performance was set at 80% correct responses (20 out of 25) as indicated in the description of the matching tests on auditory abilities performed by Bech and Zacharov (Bech & Zacharov, 2006).

**Screening Task 2: Localization of the Sound Source**

The second screening task was conducted to verify the participant's ability to detect the sound source. The participant was positioned in the central listening position of the silent audiometric cabin. Five different acoustic stimuli (Table 3), 500 ms long, were created and played simultaneously on five channels of the Surround sound system (LFE channel excluded). The stimuli consisted of a background pink noise sampled at 48000 Hz reproduced in four channels and a pure 1000 Hz cue tone reproduced by only one channel in each stimulus (Table S3). Loudness difference between background noise (-48 LUFS) and cue tone (-23 LUFS) was set at a -25 LUFS. Each stimulus was repeated four times, for a total of 20 trials.

| **Stimulus** | ***“Ls”*** | ***“L”*** | ***“C”*** | ***“R”*** | ***“Rs*”** |
| --- | --- | --- | --- | --- | --- |
| 1 | Cue | Background | Background | Background | Background |
| 2 | Background | Cue | Background | Background | Background |
| 3 | Background | Background | Cue | Background | Background |
| 4 | Background | Background | Background | Cue | Background |
| 5 | Background | Background | Background | Background | Cue |

**Table S3.** Localization of the Sound Source Screening Test Stimuli.

The participants were given a forced-choice task, in which they had to listen to the stimulus and indicate the origin of the cue tone. At the end of each playback, the question "Which speaker reproduced the *beep*?" was displayed on the screen and the participant had to answer, within 5 s, by choosing from the options shown in Figure S2.


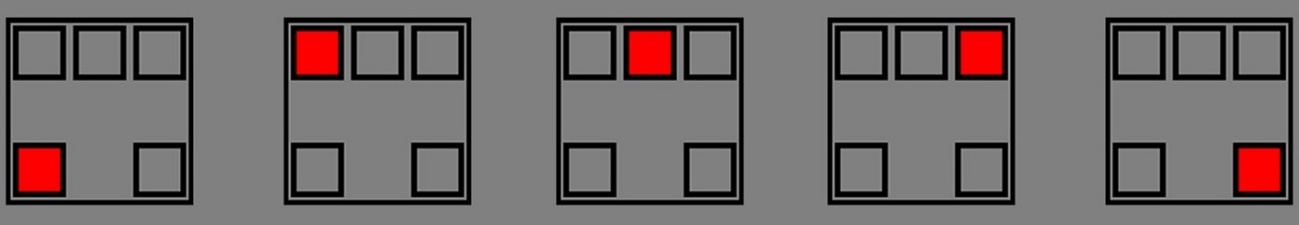


**Figure S2.** Location Maps (from left to right) for “Ls”, “L”, “C”, “R”, “Rs” channels.

The cut-off criterion for participants’ performance was set at 80% correct responses (16 out of 20) based on the results of sound source location discrimination reported by Lopatka and colleagues (Lopatka et al., 2016).

**Experiment 1**

**Participants**

Questionnaires descriptive statistics are shown in Table S4.

| **Questionnaire** | **Min.** | **Max.** | **M** | **SD** |
| --- | --- | --- | --- | --- |
| BMEQ C | 13 | 28 | 19.3 | 4.8 |
| BMEQ I | 7 | 33 | 20.0 | 7.1 |
| BMEQ S | 8 | 18 | 12.2 | 2.8 |
| BMEQ A | 36 | 50 | 43.3 | 3.5 |
| BMEQ P | 34 | 71 | 58.5 | 11.1 |
| BMEQ R | 19 | 45 | 35.2 | 6.3 |
| IRI CE | 3 | 13 | 7.4 | 3.6 |
| IRI DP | 11 | 26 | 17.7 | 4.8 |
| IRI PT | 0 | 25 | 8.6 | 5.6 |
| IRI FS | 0 | 18 | 8.1 | 4.5 |
| ITQ | 46 | 94 | 73.8 | 13.4 |
| VMIQ-2 EVI | 12 | 48 | 26.8 | 10.3 |
| VMIQ-2 IVI | 12 | 55 | 27.8 | 13.2 |
| VMIQ-2 KIN | 12 | 55 | 26.8 | 13.0 |
| F-IEQ CAP | 44 | 77 | 62.8 | 10.6 |
| F-IEQ DIS | 10 | 20 | 15.4 | 2.6 |
| F-IEQ COM | 9 | 27 | 15.5 | 4.5 |
| F-IEQ TRA | 16 | 27 | 23.3 | 3.3 |

**Table S4.** Questionnaire Descriptive Statistics (Min.= minimum score, Max.=maximum score, M=mean, SD=tandard deviations). B-MEQ: Brief Music Experience Questionnaire (C: Commitment to music, I: Innovative Musical Aptitude, S: Social uplift, A: Affective reactions, P: Positive psychotropic effects, R: Reactive musical behavior); IRI: Interpersonal Reactivity Index (PT: Perspective Taking, FS; Fantasy, CE: Empathic Concern, DP: Personal Distress); ITQ: Immersive Tendencies Questionnaire; VMIQ-2: Vividness of Movement Imagery Questionnaire-2 (EVI: External self Visual Imagery, IVI: Internal first-person Visual Imagery, KIN: Kinesthetic Imagery); F-IEQ: Film Immersive Experience Questionnaire (CAP: Captivation, DIS: Real-world dissociation, COM: Comprehension, TRA: Transportation).

**Behavioral Questions**

The explicit questions formulated by the authors and proposed to the participants aim to measure four potential dimensions of sense of Presence capturing subjective Enjoyment, Emotional Involvement, Physical Immersion, and perceived Realism, allowing us to characterize the spatialized sound experience and to better understand the participants' experience within the cinematic content.

1. Enjoyment (EN): "How much did you like the scene?"

This question assesses the subjective enjoyment experienced by the participants. It focuses on their overall liking or preference for the reproduced scene. By measuring enjoyment, we could also gauge the extent to which the participants found the acoustic reproduction entertaining or satisfying.

1. Emotional Involvement (EI): "How much did you feel emotionally involved?"

This question addresses the emotional impact of the reproduced scene. It helps understand how the acoustic scene resonates with participants on an emotional level, providing valuable information about its effectiveness in evoking emotional responses.

1. Physical Immersion (PI): "How much did you feel physically immersed?

This question focuses on the participants' sense of physical immersion in the reproduced scene. Hence, it aims to assess the extent to which the participants felt physically present or embodied within the mediated acoustic environment. Physical immersion can be related to sensory experiences and can involve aspects such as spatial presence, hapticity, or a feeling of being "transported" into the scene.

1. Realism (RE): "How realistic did you judge the scene?"

This question addresses the perceived realism of the reproduced scene. It prompts participants to evaluate how believable or authentic was the acoustic reproduction. Realism is an important aspect of the sense of Presence, as it contributes to the participants' ability to suspend disbelief and perceive the mediated acoustic environment as plausible or authentic.

**Experiment 2**

**Participants**

Questionnaires descriptive statistics are shown in Table S5.

| **Questionnaire** | **Min.** | **Max.** | **M** | **SD** |
| --- | --- | --- | --- | --- |
| BMEQ C | 17 | 23 | 18.3 | 2.3 |
| BMEQ I | 15 | 32 | 21 | 6.4 |
| BMEQ S | 9 | 15 | 13.3 | 2.5 |
| BMEQ A | 34 | 49 | 41.4 | 3.5 |
| BMEQ P | 33 | 65 | 52.4 | 10.1 |
| BMEQ R | 17 | 43 | 33.2 | 5.3 |
| IRI CE | 5 | 14 | 5.6 | 3.8 |
| IRI DP | 13 | 23 | 15.2 | 3.7 |
| IRI PT | 2 | 20 | 7.5 | 4.4 |
| IRI FS | 3 | 17 | 6.9 | 4.1 |
| ITQ | 50 | 91 | 69.5 | 11.3 |
| VMIQ-2 EVI | 14 | 51 | 24.3 | 9.9 |
| VMIQ-2 IVI | 13 | 55 | 25.1 | 11.3 |
| VMIQ-2 KIN | 15 | 54 | 26.4 | 12.1 |
| F-IEQ CAP | 45 | 70 | 63.8 | 9.8 |
| F-IEQ DIS | 11 | 21 | 12.1 | 2.1 |
| F-IEQ COM | 13 | 22 | 13.4 | 4.1 |
| F-IEQ TRA | 11 | 24 | 22.3 | 2.3 |

**Table S5.** Questionnaire Descriptive Statistics (Min.= minimum score, Max.=maximum score, M=mean, SD=tandard deviations). B-MEQ: Brief Music Experience Questionnaire (C: Commitment to music, I: Innovative Musical Aptitude, S: Social uplift, A: Affective reactions, P: Positive psychotropic effects, R: Reactive musical behavior); IRI: Interpersonal Reactivity Index (PT: Perspective Taking, FS; Fantasy, CE: Empathic Concern, DP: Personal Distress); ITQ: Immersive Tendencies Questionnaire; VMIQ-2: Vividness of Movement Imagery Questionnaire-2 (EVI: External self Visual Imagery, IVI: Internal first-person Visual Imagery, KIN: Kinesthetic Imagery); F-IEQ: Film Immersive Experience Questionnaire (CAP: Captivation, DIS: Real-world dissociation, COM: Comprehension, TRA: Transportation).

**Results**

**Alpha Band Range**

**
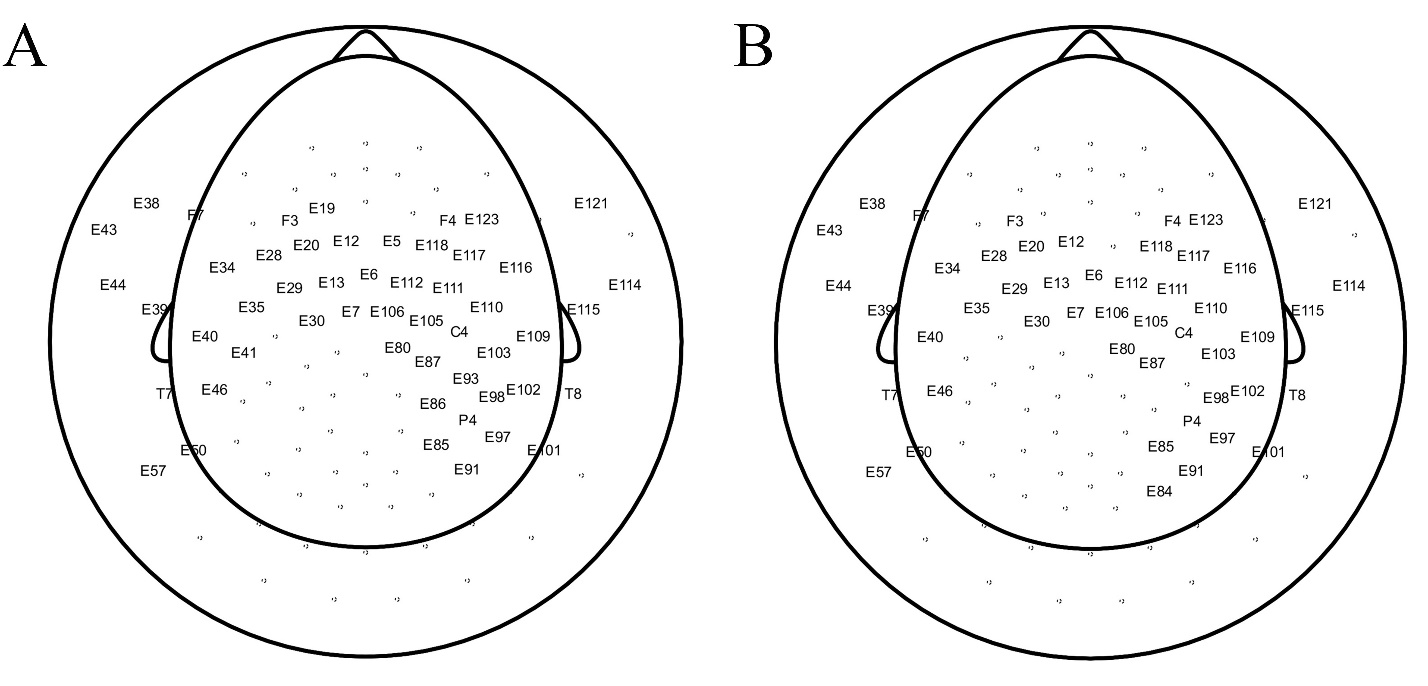
**

Figure S3 **(A)** Surround - Monophonic Alpha band (8 – 10 Hz) cluster channels (peak time 5s from stimulus onset). **(B)** Surround - Control Alpha band (8 – 10 Hz) cluster channels (peak time 5s from stimulus onset).

**Low Beta Band Range**

**
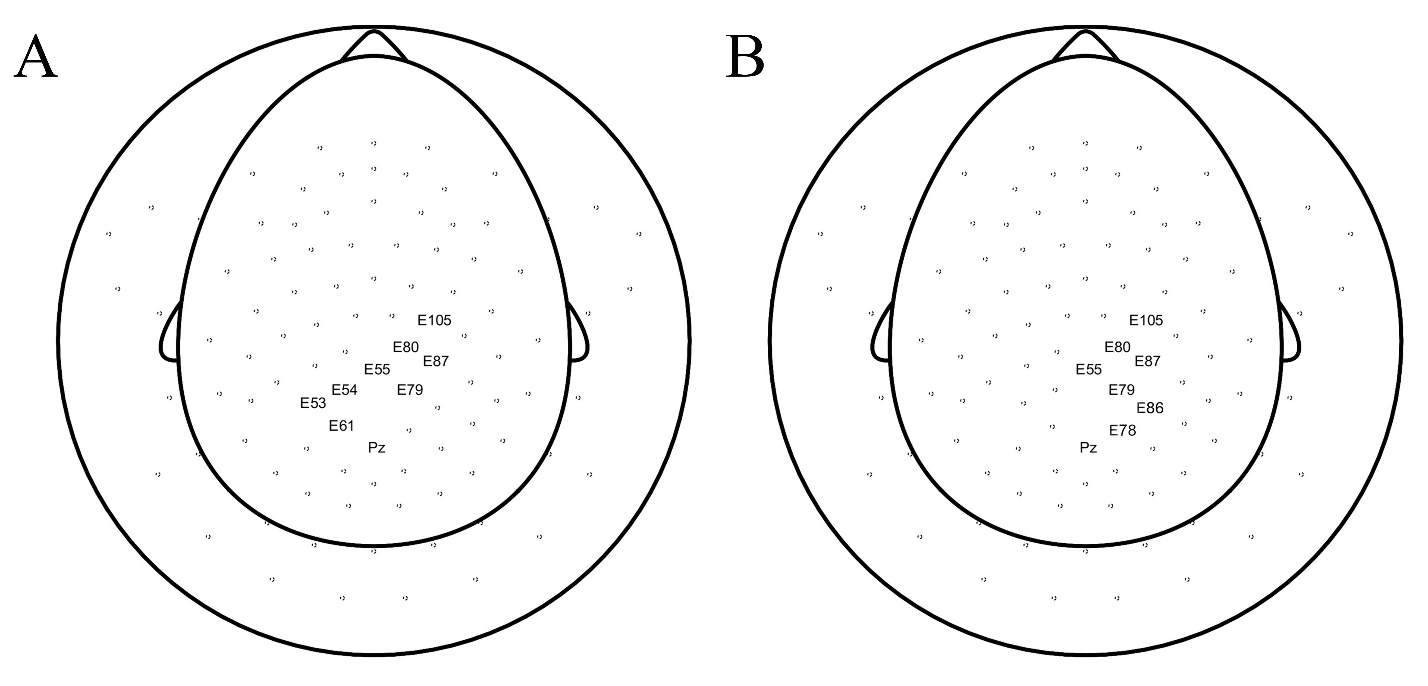
**

Figure S4 **(A)** Surround - Monophonic Low Beta band (16 – 18 Hz) cluster channels (peak time 4.5s from stimulus onset). **(B)** Surround - Control Low Beta band (16 – 18 Hz) cluster (peak time 4.5s from stimulus onset) channels.

**Behavioral Analysis**

In order to investigate whether Physical Immersion (PI) is modulated by Presentation modes, a linear mixed effect analysis was performed, respectively. Following a hierarchical approach, we initially created a simple model using one parameter, and we progressively added others with the aim to evaluate whether their inclusion improved model fit. Likelihood ratio tests, Akaike Information Criterion (AIC) and Bayesan Information Criterion (BIC) were used to were used to rigorously choose which parameters improved model fit. We entered participants’ scores (PI) as dependent variable and Presentation mode (4 levels: Monophonic, Surround, Monophonic Control, Surround Control) as independent fixed variables. The participants were included as a random intercept and Presentation mode as a random slope. This approach accounted for the within-subject and between-subject variability in the data. Outliers were identified and excluded from the analysis based on the standardized model residuals and a threshold value of Cook's distance (threshold=1). Post-hoc tests were conducted using Tukey's correction for multiple comparisons and Kenward-Roger degrees-of-freedom approximation method.

**Behavioral Results**

The model explained 98% of the variance in dependent variable, taking into account the random effects (R^2^_m_ = 0.82; R^2^_c_ = 0.98). The model revealed a significant main effect of Presentation modes (F_(3, 13.88)_ = 61.36, *p* < .001), showing that participants attributed significantly higher absolute scores when stimuli were presented in the Surround Presentation mode than when they were presented in the Monophonic Presentation mode (t_(14)_ = 6.73, *p* < .001), in Monophonic Control Presentation mode (t_(14)_ = 13.4, *p* < .001) or in the Surround Control Presentation mode (t_(14)_ = 10.59, *p* < .001) (Surround: M = 76.38, CIs = 69.17, 83.6; Surround Control: M = 30.67, CIs = 24.41, 36.93; Monophonic: M = 69.95, Cis = 62.66, 77.25; Monophonic Control: M = 17.72, CIs = 12.11, 23.33). In addition, participants attributed significantly higher scores when stimuli were presented in the Monophonic Presentation mode than when they were presented in Monophonic Control Presentation mode (t_(14)_ = 12.62, *p* < .001) or in the Surround Control Presentation mode (t_(14)_ = 8.97, *p* < .001). Interestingly, attributed significantly higher scores when stimuli were presented in the Surround Control Presentation mode than when they were presented in the Monophonic Control Presentation mode (t_(14)_ = 5.21, *p* < .001).

**References**

Albiero, P., Ingoglia, S., & Cocco, A. L. (2006). Contributo all’adattamento italiano dell’Interpersonal Reactivity Index. *TESTING PSICOMETRIA METODOLOGIA*, *13*(2), 107–125.

Bech, S., & Zacharov, N. (2006). *Perceptual Audio Evaluation–Theory, Method and Application*. <https://doi.org/10.1002/9780470869253>

Bech, S., & Zacharov, N. (2018). *Perceptual Audio Evaluation–Theory, Method and Application*. <https://doi.org/10.1002/9780470869253>

Brainard, D. H. (1997). The Psychophysics Toolbox. *Spatial Vision*, *10*, 433–436.

EBU. (2014). Recommendation 128, Loudness normalisation and permitted maximum level of audio signals. *European Broadcasting Union*.

Fernández-Aguilar, L., Navarro-Bravo, B., Ricarte, J., Ros, L., & Latorre, J. M. (2019). How effective are films in inducing positive and negative emotional states? A meta-analysis. *PLOS ONE*, *14*(11), e0225040. <https://doi.org/10.1371/journal.pone.0225040>

Figueiredo, H. F., Bodie, B. L., Tauchi, M., Dolgas, C. M., & Herman, J. P. (2003). Stress Integration after Acute and Chronic Predator Stress: Differential Activation of Central Stress Circuitry and Sensitization of the Hypothalamo-Pituitary-Adrenocortical Axis. *Endocrinology*, *144*(12), 5249–5258. <https://doi.org/10.1210/en.2003-0713>

ISO. (2017). Standard 7029:2017, Statistical distribution of hearing thresholds related to age and gender. *International Organization for Standardization*.

Larsen, E., Iyer, N., Lansing, C. R., & Feng, A. S. (2008). On the minimum audible difference in direct-to-reverberant energy ratioa). *The Journal of the Acoustical Society of America*, *124*(1), 450–461. <https://doi.org/10.1121/1.2936368>

Latinus, M., & Belin, P. (2011). Human voice perception. *Current Biology*, *21*(4), R143–R145. <https://doi.org/10.1016/j.cub.2010.12.033>

Leppänen, J. M., & Nelson, C. A. (2009). Tuning the developing brain to social signals of emotions. *Nature Reviews Neuroscience*, *10*(1), 37–47. <https://doi.org/10.1038/nrn2554>

Lopatka, K., Kotus, J., & Czyzewski, A. (2016). Detection, classification and localization of acoustic events in the presence of background noise for acoustic surveillance of hazardous situations. *Multimedia Tools and Applications*, *75*(17), 10407–10439. <https://doi.org/10.1007/s11042-015-3105-4>

MathWorks. (2021). *Statistics and Machine Learning Toolbox Documentation, Natick, Massachusetts: The MathWorks Inc.* <https://www.mathworks.com/help/stats/index.html>

Mattila, V.-V., & Zacharov, N. (2001). Generalized listener selection (GLS) procedure. *AES 110th CONVENTION*.

Palan, S., & Schitter, C. (2018). Prolific.ac—A subject pool for online experiments. *Journal of Behavioral and Experimental Finance*, *17*, 22–27. <https://doi.org/10.1016/j.jbef.2017.12.004>

Peirce, J., Gray, J. R., Simpson, S., MacAskill, M., Höchenberger, R., Sogo, H., Kastman, E., & Lindeløv, J. K. (2019). PsychoPy2: Experiments in behavior made easy. *Behavior Research Methods*, *51*(1), 195–203. <https://doi.org/10.3758/s13428-018-01193-y>

Rawashdeh, S. (2021). *Frequency Response of the Ear , Hearing Test. MATLAB Central File Exchange.* <https://www.mathworks.com/matlabcentral/fileexchange/16101-frequency-response-of-the-ear-hearing-test>

Roberts, R., Callow, N., Hardy, L., Markland, D., & Bringer, J. (2008). Movement Imagery Ability: Development and Assessment of a Revised Version of the Vividness of Movement Imagery Questionnaire. *Journal of Sport and Exercise Psychology*, *30*(2), 200–221. <https://doi.org/10.1123/jsep.30.2.200>

Sauter, M., Draschkow, D., & Mack, W. (2020). Building, Hosting and Recruiting: A Brief Introduction to Running Behavioral Experiments Online. *Brain Sciences*, *10*(4), 251. <https://doi.org/10.3390/brainsci10040251>

Visch, V. T., Tan, E. S., & Molenaar, D. (2010). The emotional and cognitive effect of immersion in film viewing. *Cognition and Emotion*, *24*(8), 1439–1445. <https://doi.org/10.1080/02699930903498186>

Werner, P. D., Swope, A. J., & Heide, F. J. (2006). The Music Experience Questionnaire: Development and Correlates. *The Journal of Psychology*, *140*(4), 329–345. <https://doi.org/10.3200/jrlp.140.4.329-345>

Witmer, B. G., & Singer, M. J. (1998). Measuring Presence in Virtual Environments: A Presence Questionnaire. *Presence: Teleoperators and Virtual Environments*, *7*(3), 225–240. <https://doi.org/10.1162/105474698565686>

Wöllner, C., Hammerschmidt, D., & Albrecht, H. (2018). Slow motion in films and video clips: Music influences perceived duration and emotion, autonomic physiological activation and pupillary responses. *PLoS ONE*, *13*(6), e0199161. <https://doi.org/10.1371/journal.pone.0199161>

World Medical Association. (2013). World Medical Association Declaration of Helsinki: Ethical Principles for Medical Research Involving Human Subjects. *JAMA*, *310*(20), 2191–2194. <https://doi.org/10.1001/jama.2013.281053>
